# SGLT-2 inhibitors improve cardiac function in hypertrophic cardiomyopathy: a real-world propensity score-matched study

**DOI:** 10.3389/fcvm.2026.1742682

**Published:** 2026-02-12

**Authors:** Cong Ding, Fangchao Lv, Lin Wang, Xiaohong Xu

**Affiliations:** 1 Department of Gastroenterology, Affiliated Hangzhou First People’s Hospital, School of Medicine, Westlake University, Hangzhou, Zhejiang, China; 2 Department of Cardiology, Zhejiang Hospital, Hangzhou, Zhejiang, China

**Keywords:** heart failure, hypertrophic cardiomyopathy, left ventricular diastolic function, real-world study, sodium-glucose cotransporter 2 inhibitor

## Abstract

**Background and aims:**

Hypertrophic cardiomyopathy (HCM) is characterized by left ventricular hypertrophy and diastolic dysfunction. While sodium-glucose cotransporter 2 inhibitors (SGLT-2i) have demonstrated efficacy in heart failure (HF), their role in HCM remains underexplored. This real-world study aimed to evaluate the clinical efficacy of SGLT-2i in HCM patients.

**Methods and results:**

A retrospective analysis was conducted on HCM patients admitted between January 2021 and December 2024. After PSM, 94 patients initiating SGLT-2i were compared with 94 controls. Primary endpoints included changes (Δ) in echocardiographic parameters and NYHA class at 6-month follow-up. Secondary endpoint was readmission for HF by June 2025. At 6-month follow-up, patients treated with SGLT-2i showed significantly greater improvements in key parameters compared to controls: septal *e*′ (Δ 0.7 ± 1.3 vs. Δ 0.04 ± 1.6 cm/s, *p* = 0.002), *E*/*e*′ (Δ −5.1 ± 8.7 vs. Δ 0.4 ± 6.4, *p* < 0.001), and IVST (Δ −1.3 vs. Δ −0.2 mm, *p* = 0.005), alongside a greater reduction in NYHA class [−1 (−1 to −0.25) vs. −1 (−1 to 0), *p* = 0.031]. Multivariate analysis confirmed sustained differences in improvements of septal *e*′ (*t* = 2.26, *p* = 0.025), *E*/*e*′ (*t* = −3.75, *p* < 0.001) and NYHA class (*p* = 0.038). No significant difference was found in HF readmission (20 events in SGLT-2i group vs. 17 in control group; log-rank *p* = 0.73) after 16.3-month median follow-up. No hypoglycemic events occurred and there was no significant deterioration in renal function.

**Conclusion:**

SGLT-2i administration was associated with improved left ventricular diastolic function and NYHA class in HCM patients without increasing risks of renal dysfunction or hypoglycemia, supporting its potential therapeutic value in this population.

## Introduction

Hypertrophic cardiomyopathy (HCM) is a hereditary cardiomyopathy characterized by left ventricular (LV) hypertrophy, often accompanied by diastolic dysfunction, myocardial fibrosis and microvascular dysfunction (1–4). Its prevalence is estimated at 1:500 in the general population, and may reach 1:200 when undiagnosed or asymptomatic cases are considered, highlighting its substantial clinical and public health impact (1).

Conventional treatment strategies for HCM have focused on symptom control and risk reduction through β-blockers, calcium channel blockers, and invasive procedures. Septal reduction therapy (SRT) is reserved for patients with significant left ventricular outflow tract obstruction (LVOTO), whereas implantable cardioverter-defibrillators (ICDs) are employed for preventing sudden cardiac death both primarily and secondarily (2–4). Although advances in understanding have reduced annual mortality from 6% to approximately 0.5%—now comparable to the general population (1)—many patients continue to experience impaired quality of life due to dynamic LVOTO, diastolic impairment, arrhythmias, and progression to HF (1), emphasizing the demand for targeted therapies.

Mavacamten, a novel cardiac myosin inhibitor, represents a therapeutic breakthrough by specifically attenuating LVOTO and postponing SRT in suitable candidates (5, 6). Despite its clinical benefits, widespread use is limited by high costs and restricted availability.

Sodium-glucose cotransporter 2 inhibitor (SGLT-2i) is originally used to treat diabetes mellitus (DM) through inhibiting the reabsorption of glucose by the epithelial cells of the renal proximal tubule, increasing the excretion of glucose in the urine, and thus lowering blood sugar (7). Apart from glycemic benefits, SGLT-2i have exhibited considerable cardioprotective properties in heart failure patients—including those with reduced or preserved ejection fraction (HFrEF and HFpEF)— regardless of DM (8, 9). Mechanistic studies suggest these drugs improve myocardial energetics, reduce oxidative stress and inflammatory responses, suppress fibrotic processes, promote reverse remodeling, and lessen calcium accumulation (10). Given that these actions correspond to central pathological features of HCM, SGLT-2i may offer potential in enhancing diastolic performance and alleviating hypertrophy and fibrosis (3).

Notably, pivotal randomized controlled trials (RCTs) establishing the cardiovascular benefits of SGLT-2i systematically excluded patients with HCM (11, 12). Consequently, the current evidence guiding their use in HCM is extrapolated from studies in HFpEF, a population with distinct pathophysiology. This has created a critical evidence gap regarding the efficacy of SGLT-2i on HCM-specific pathological features, such as left ventricular hypertrophy and diastolic dysfunction. To address this gap, we conducted this real-world, propensity score-matched (PSM) study with two primary objectives: first, to evaluate the effects of SGLT-2i on echocardiographic markers of cardiac structure and diastolic function specific to HCM; and second, to assess their safety and preliminary impact on functional status in this understudied population.

## Methods

### Study population

This was a single-center retrospective cohort study. We searched inpatients from January 2021 to December 2024 from electronic medical records in our department. Patients were included meeting the following criteria: ① diagnosed with HCM (3), which was defined echocardiographically as a maximal left ventricular wall thickness ≥15 mm in the absence of other cardiac or systemic diseases that could account for the hypertrophy; ② ≥18 years old; ③ patients with both obstructive (left ventricular outflow tract pressure gradient ≥30 mmHg) and non-obstructive forms of HCM were eligible for inclusion.

Exclusion criteria were as follows: ① previously used SGLT-2i; ② combined with severe renal insufficiency (eGFR <30 mL/min/1.73 m^2^); ③ echocardiographic data were not available at 6 (±1) months after discharge; ④ STR was performed during the follow-up period.

Given that this was a retrospective observational study and no patient intervention was required, informed consent from subjects could be waived, which was approved by the ethics committee. In conducting the study, we maintained a strong focus on the protection of patients’ personal interests, rights, privacy, and image rights.

#### Group assignment

Based on SGLT-2i exposure, patients selected were classified to two groups: the SGLT-2i group (newly initiated during the admission) and the control group (no SGLT-2i use). Medication history was obtained from electronic prescription records.

### Outcomes

Our primary endpoints were the echocardiographic parameters and NYHA class, the former including mitral annular tissue velocity (*e*′), early diastolic mitral inflow velocity (*E*)/*e*′, and interventricular septal thickness (IVST) at 6-month (±1 month) follow-up.

Secondary endpoint was unplanned HF readmission (based on ICD-10 codes) by June 2025. Hospitalizations where HF was not the primary driver (e.g., for arrhythmia without acute HF, pneumonia, or elective surgery) were excluded. This endpoint was adjudicated by two independent cardiologists (F.L and L.W).

Safety endpoints included adverse reactions such as hypoglycemia and urinary tract infection.

### Data collection

Baseline data from HCM patients were collected, including medical history, concomitant medications, laboratory parameters—such as serum creatinine, HbA1c, fasting plasma glucose (FPG), B-type natriuretic peptide (BNP)—as well as echocardiographic measures at baseline and 6 months (±1 month) after discharge. We also recorded endpoint events including HF readmission, hypoglycemia and urinary tract infection.

For patients not followed up at our institution, telephone follow-up was made to obtain follow-up information from other healthcare facilities and to ascertain whether endpoint events had occurred. Those without any post-discharge follow-up data were excluded from the study.

### Statistical analysis

We employed 1:1 PSM to balance baseline characteristics between the groups. The propensity score model incorporated clinically relevant confounding factors, including sex, age, body mass index (BMI), history of hypertension, and DM. Matching was performed using a 1:1 nearest-neighbor algorithm with a caliper width set to 0.2 standard deviations of the logit propensity score. Covariate balance was assessed by calculating absolute standardized mean differences (SMD) for all baseline variables.

Numbers (frequencies) were used to describe categorical variables, mean ± standard deviation to describe normal distribution continuous variables while median (interquartile range, IQR) to describe non-normal distribution. *χ*2 test was used for comparison between categorical variables, while T-test was used for normally distributed continuous variables and Mann–Whitney U test for non-normal continuous variables. Multivariable analysis was performed using linear regression or a bootstrap multiple linear regression model (1,000 repetitions), depending on whether the continuous variables were normally distributed or not. All fitted models were subjected to diagnostic checks, which confirmed the adherence to key statistical assumptions: residual independence (Durbin-Watson statistic), homoscedasticity, and approximate normality of residuals. Furthermore, the absence of significant multicollinearity was verified (all variance inflation factors <5). Model fit was reported using the adjusted *R*^2^.

Kaplan–Meier curve was used to evaluate differences in HF readmission events. We also performed subgroup analysis of obstructive hypertrophic cardiomyopathy (oHCM) and non-obstructive hypertrophic cardiomyopathy (noHCM) at low-risk.

We used IBM SPSS version 26.0 (SPSS Inc., Chicago, IL, USA) and R version 4.3.1 (R Foundation for Statistical Computing, Vienna, Austria) to perform the statistical analyses described above.

## Results

### Baseline characteristics

#### Full cohort

**Figure 1:**
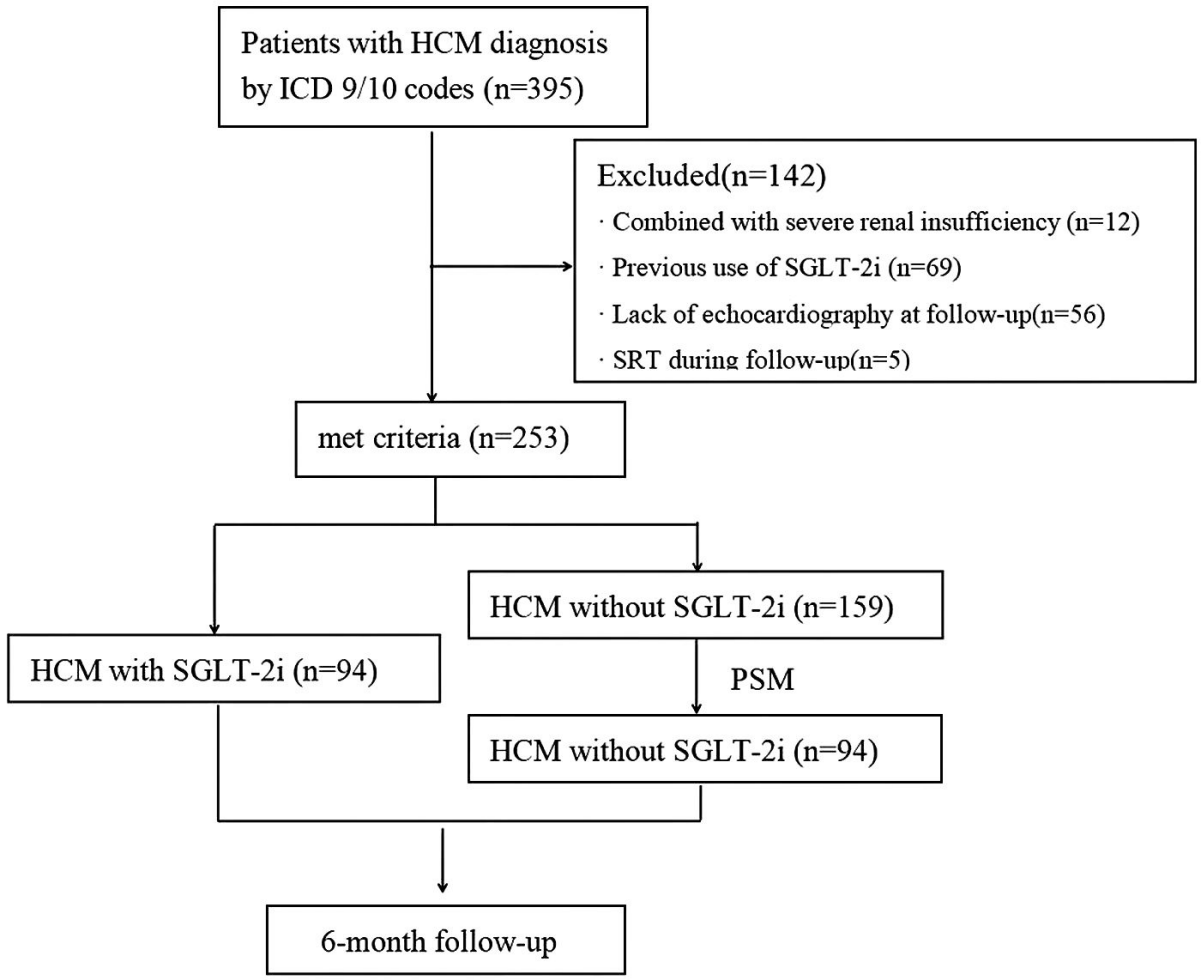
Flowchart depicting the participant selection process for the study. HCM, hypertrophic cardiomyopathy; ICD, international Classification of diseases; SGLT-2i, sodium-glucose cotransporter-2 inhibitors; SRT, Septal reduction therapy; PSM, propensity score matching.

A total of 395 patients with HCM were enrolled from January 2021 to December 2024. After excluding 142 individuals based on the criteria outlined in Figure 1, 253 eligible patients were included in the analysis. Among them, 163 (64.4%) had hypertension, 96 (37.9%) had DM, and 148 (58.4%) were diagnosed with HF. The mean age was 65.0 ± 14.7 years, and 67.2% (170/253) were male. Baseline characteristics are presented in Table 1. Within the full cohort, 94 patients (37.2%) received SGLT-2i. Compared to non-users, those treated with SGLT-2i were generally older and showed a higher prevalence of hypertension, DM, HF, and AF. They also had elevated baseline values of BNP, HbA1c, FPG, septal *e*′, and *E*/*e*′.

**Table 1:** Baseline characteristics for matched study population.

Parameter	Full cohort			PSM cohort			
	SGLT-2i (+) *n* = 94	SGLT-2i (−) *N* = 159	*p* value	SGLT-2i (+) *n* = 94	SGLT-2i (−) *n* = 94	*p* value	SMD
Age, years	69.0 ± 14.0	64.0 ± 14.7	0.007	69.0 ± 14.0	65.4 ± 14.8	0.092	0.24
Male sex, *n* (%)	62 (66.0%)	108 (67.9%)	0.75	62 (66.0%)	65 (69.1%)	0.64	0.074
Smoking, *n* (%)	49 (52.1%)	75 (47.2%)	0.45	49 (52.1%)	43 (45.7%)	0.38	0.13
BMI, kg/m^2^^a^	23.2 (21.9 to 26.1)	24.1 (20.5 to 26.4)	0.28	23.2 (21.9 to 26.1)	23.6 (21.8 to 25.8)	0.77	0.13
Blood pressure, mmHg							
Systolic	131.8 ± 21.7	139.0 ± 25.2	0.048	131.8 ± 21.7	137.5 ± 24.9	0.096	0.24
Diastolic	74.9 ± 15.8	78.5 ± 17.4	0.13	74.9 ± 15.8	78.0 ± 16.7	0.19	0.19
HR, bpm	74.9 ± 15.3	75.7 ± 16.3	0.76	74.9 ± 15.3	76.6 ± 16.4	0.46	0.11
NYHA class			0.001			0.16	0.28
NYHA II	35 (37.2%)	95 (59.7%)	–	35 (37.2%)	48 (51.1%)	–	–
NYHA III	48 (51.1%)	46 (28.9%)	–	48 (51.1%)	38 (40.4%)	–	–
NYHA IV	11 (11.7%)	18 (11.3%)	–	11 (11.7%)	8 (8.5%)	–	–
Comorbidities							
Hypertension, *n* (%)	68 (72.3%)	95 (59.7%)	0.043	68 (72.3%)	71 (75.5%)	0.64	0.078
CAD, *n* (%)	39 (41.5%)	60 (37.7%)	0.75	39 (41.5%)	37 (39.4%)	0.77	0.064
DM, *n* (%)	46 (48.9%)	50 (31.4%)	0.006	46 (48.9%)	40 (41.7%)	0.42	0.13
HF, *n* (%)	75 (79.8%)	73 (45.9%)	<0.001	75 (79.8%)	59 (62.8%)	0.01	0.37
Af, *n* (%)	45 (53.2%)	49 (30.9%)	0.007	45 (53.2%)	36 (38.3%)	0.19	0.20
Stroke, *n* (%)	20 (21.3%)	21 (13.2%)	0.12	20 (21.3%)	16 (17.0%)	0.46	0.11
CKD, *n* (%)	30 (31.9%)	38 (23.9%)	0.18	30 (31.9%)	26 (27.7%)	0.53	0.09
COPD, *n* (%)	6 (6.4%)	6 (3.8%)	0.38	6 (6.4%)	3 (3.2%)	0.31	0.15
Laboratory data							
Hemoglobin, mg/dL	136.3 ± 19.2	137.6 ± 19.6	0.63	136.3 ± 19.2	134.4 ± 18.4	0.48	0.10
Creatinine, mg/dL	95.1 ± 23.4	86.9 ± 36.9	0.018	95.1 ± 23.4	91.7 ± 35.1	0.44	0.11
BNP, pg/mL^a^	446.0 (289.0 to 1,007.1)	110.0 (10.0 t o870.3)	<0.001	446.0 (289.0 to 1,007.1)	209.3 (55.5 to 565.3)	0.005	0.51
FPG, mmol/L	6.7 ± 2.6	5.9 ± 2.0	0.006	6.7 ± 2.6	6.2 ± 2.1	0.18	0.21
HbA1c, %	6.7 ± 1.6	6.1 ± 1.1	<0.001	6.7 ± 1.6	6.6 ± 1.3	0.71	0.069
SUA, mg/dL	413.2 ± 104.8	401.8 + 105.6	0.41	413.2 ± 104.8	408.6 ± 98.1	0.76	0.14
LDL, mmol/L	2.4 ± 0.9	2.7 ± 1.0	0.022	2.4 ± 0.9	2.5 ± 1.0	0.66	0.11
Echocardiographic parameters							
IVST, mm^a^	18.2 (15.0 to 21.7)	16.4 (13.0 to 20.6)	0.20	18.2 (15.0 to 21.7)	16.0 (14.0 to 19.6)	0.18	0.48
LAD, mm	48.3 ± 7.9	40.7 ± 10.1	<0.001	48.3 ± 7.9	42.2 ± 7.3	0.013	0.80
LVEDD, mm	47.2 ± 7.5	47.1 ± 6.6	0.90	47.2 ± 7.5	46.2 ± 7.3	0.37	0.14
LVEF, %	57.8 ± 10.3	61.0 ± 10.7	0.016	57.8 ± 10.3	60.2 ± 6.4	0.007	0.28
Septal *e*′	4.2 ± 1.4	5.0 ± 1.6	<0.001	4.2 ± 1.4	4.8 ± 1.3	0.004	0.44
*E*/*e*′	19.2 ± 9.5	14.9 ± 7.6	<0.001	19.2 ± 9.5	14.4 ± 5.7	<0.001	0.61
LVOTPG at rest^a^	6.0 (3.0 to 12.0)	7.0 (3.0 to 13.0)	0.38	6.0 (3.0 to 12.0)	6.0 (4.0 to 11.5)	0.43	1.15
LVOT obstruction, *n* (%)	11 (11.7%)	18 (11.3%)	0.91	11 (11.7%)	13 (13.8%)	0.66	0.062
Therapy							
Beta-Blocker, *n* (%)	69 (73.4%)	122 (76.7%)	0.59	69 (73.4%)	76 (80.9%)	0.23	0.18
ACEI/ARB/ARNI, *n* (%)	71 (75.5%)	98 (61.6%)	0.035	71 (75.5%)	69 (73.4%)	0.74	0.05
ARNI, *n* (%)	63 (67.0%)	78 (49.1%)	0.003	63 (67.0%)	59 (62.8%)	0.54	0.09
SGLT2-i, *n* (%)	94 (100%)	–	NA	94 (100%)	–	NA	NA
Dapagliflozin	69 (73.4%)	–	NA	69 (73.4%)	–	NA	NA
Empagliflozin	19 (20.2%)	–	NA	19 (20.2%)	–	NA	NA
Others	6 (6.4%)	–	NA	6 (6.4%)	–	NA	NA
MRA, *n* (%)	59 (62.8%)	58 (36.5%)	<0.001	59 (62.8%)	39 (41.4%)	0.003	0.44
Loop Diuretic, *n* (%)	65 (69.1%)	78 (49.1%)	<0.001	65 (69.1%)	48 (51.1%)	0.01	0.37
CCB, *n* (%)	70 (74.5%)	116 (73.0%)	0.79	70 (74.5%)	68 (72.3%)	0.74	0.05
Antiplatelet drug, *n* (%)	40 (42.6%)	61 (38.4%)	0.51	40 (42.6%)	50 (53.2%)	0.15	0.21
OACs, *n* (%)	48 (57.4%)	57 (35.8%)	0.018	48 (51.1%)	36 (38.3%)	0.080	0.26
Statin, *n* (%)	69 (73.4%)	110 (69%)	0.48	69 (73.4%)	67 (71.3%)	0.75	0.05
Metformin, *n* (%)	18 (19.1%)	45(28.3%)	0.11	18 (19.1%)	24 (25.5%)	0.29	0.15
DPP4i, *n* (%)	8 (8.5%)	26 (16.4%)	0.083	8 (8.5%)	16 (17.0%)	0.080	0.26

SGLT-2i, sodium-glucose cotransporter-2 inhibitors; BMI, body mass index; HR, heart rate; CAD, coronary artery disease; MI, myocardial infarction; DM, diabetes mellitus; HF, heart failure; Af, atrial fibrillation; CKD, chronic kidney disease; COPD, chronic obstructive pulmonary disease; BNP, B-type natriuretic peptide; FPG, fasting plasma glucose; SUA, serum uric acid; LDL, low density lipoprotein cholesterol; IVST, Interventricular septal thickness; LAD, left atrial diameter; LVEDD, left ventricular end diastolic diameter; LVEF, left ventricular ejection fraction; E, early diastolic mitral inflow velocity; *e*′, mitral annular tissue velocity; LVOTPG, left ventricular outflow tract pressure gradient; LVOT, left ventricular outflow tract; ACEI/ARB/ARNI, angiotensin converting enzyme inhibitor/angiotensin receptor blocker/angiotensin receptor neprilysin inhibitor; MRA, mineralcorticoid recept antagonist; CCB, calcium channel blocker; OACs, oral anticoagulants; DPP4i: Dipeptidyl peptidase 4 inhibitors.

^a^ Median (IQR).

#### PSM cohort

**Figure 2:**
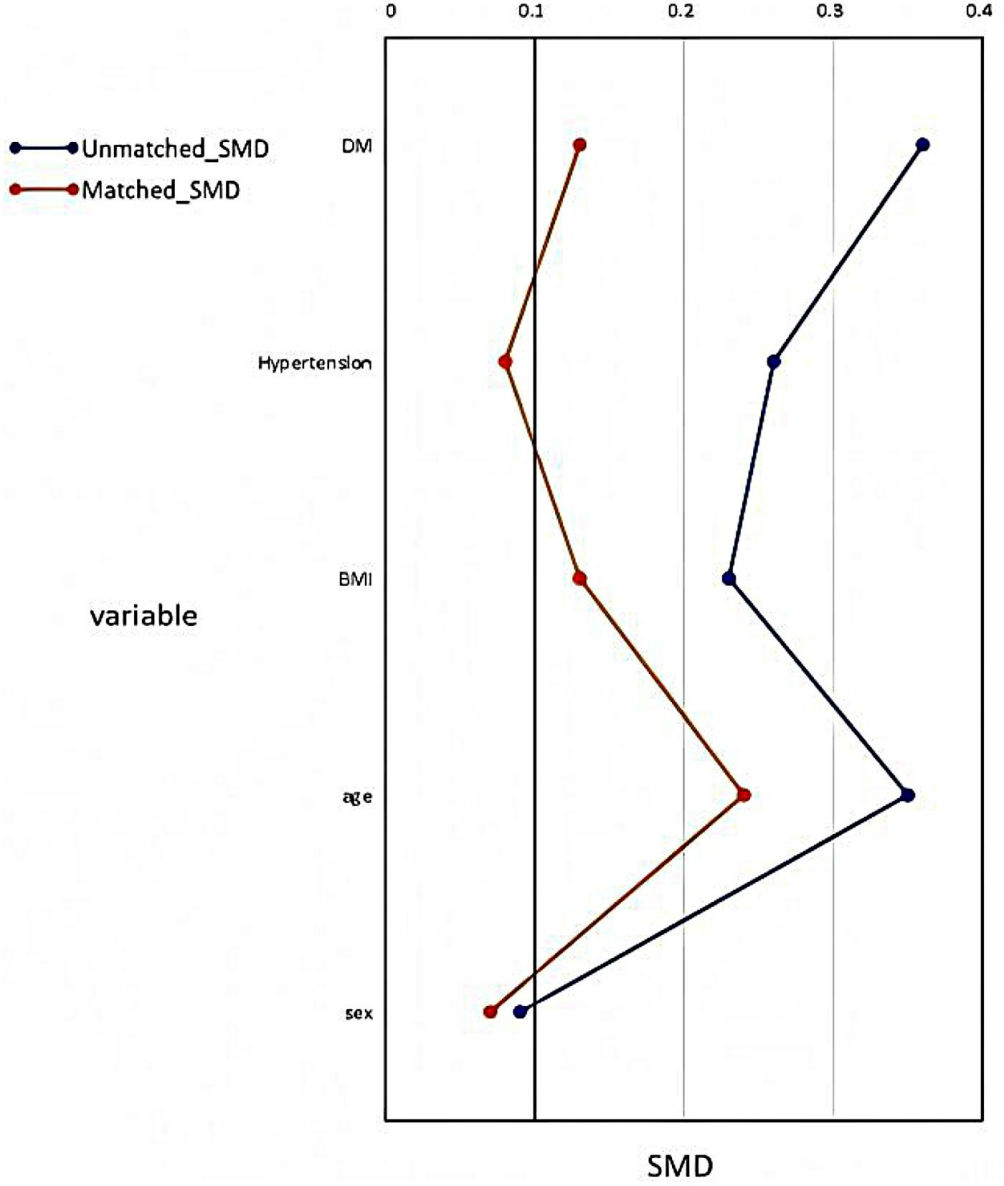
Propensity Score Matching. BMI, body mass index; SMD, Standardized mean difference.

To reduce potential confounding bias from baseline characteristics, we conducted 1:1 PSM based on sex, age, BMI, hypertension, and DM, as shown in Figure 2. A total of 94 HCM patients not using SGLT-2i were selected as controls and matched with 94 patients initiating SGLT-2i therapy. The baseline characteristics of the PSM cohort are summarized in Table 1. The cohort had a mean age of 67.2 ± 14.4 years, median BMI of 23.4 (21.3–26.2), and 67.6% were male. Comorbidities were common, including hypertension (73.9%), HF (71.3%), DM (41.5%), atrial fibrillation (AF) (45.8%), coronary artery disease (CAD) (40.4%), and chronic kidney disease (CKD) (29.8%). Median IVST was 16.2 (15.0–20.5) mm, mean LVEF was 59.0 ± 8.4%, and 12.8% of patients exhibited left ventricular outflow tract obstruction (LVOTO) at rest.

After PSM, aside from a higher prevalence of HF in the SGLT-2i group (79.8% vs. 62.8%, *p* = 0.01), no other comorbidities differed significantly between the two groups. However, baseline BNP levels, echocardiographic parameters (including LAD, LVEF, septal *e*′, and *E*/*e*′), as well as the use of mineralocorticoid receptor antagonists (MRA) and loop diuretics, still showed significant differences between groups.

### Outcomes

#### Primary endpoints

Table 2 summarizes the changes in echocardiographic parameters, laboratory indicators and NYHA class for both groups. At 6-month follow-up, in the SGLT-2i group, significant improvements from baseline were observed in septal *e*′ (4.9 ± 1.4 vs. 4.2 ± 1.4, *p* < 0.001), and *E*/*e*′ (13.9 ± 5.8 vs. 19.1 ± 9.5, *p* < 0.001). IVST, LVOTPG at rest, HR, BNP and NYHA class were significantly reduced in both groups compared to values recorded during hospitalization.

**Table 2:** Comparison of laboratory data changes at baseline and 6-month follow-up.

Parameter	SGLT-2i (+) (*n* = 94)				SGLT-2i (−) (*n* = 94)				Inter-group *p* value	Cohen’s *d*/δ
	Baseline	Follow-up	Change	*p* value	Baseline	Follow-up	Change	*p* value		
Septal *e*′	4.2 ± 1.4	4.9 ± 1.4	0.7 ± 1.3	<0.001	4.8 ± 1.3	4.8 ± 1.4	0.04 ± 1.6	0.82	0.002	0.45
*E*/*e*′	19.2 ± 9.5	13.9 ± 5.8	−5.1 ± 8.7	<0.001	14.4 ± 5.7	14.8 ± 6.0	0.4 ± 6.4	0.58	<0.001	0.72
IVST, mm	18.2 (15.0 to 21.7)	16.8 (14.2 to 19.0)	−1.3 (−3.1 to 0)	<0.001*	16.0 (14.0 to 19.6)	15.8 (13.5 to 19.0)	−0.2 (−2.0 to 0.9)	0.01*	0.005*	0.35**
LAD, mm	48.3 ± 7.9	47.2 ± 7.4	−1.1 ± 4.5	0.016	42.2 ± 7.3	42.3 ± 7.0	0.08 ± 5.5	0.89	0.11	0.24
LVEDD, mm	47.2 ± 7.5	46.9 ± 6.8	−0.3 ± 5.5	0.63	46.2 ± 7.3	46.6 ± 6.7	0.4 ± 6.5	0.56	0.45	0.12
LVEF, %	57.8 ± 10.3	59.1 ± 8.5	1.3 ± 8.8	0.14	61.2 ± 6.4	64.1 ± 7.2	2.9 ± 6.7	<0.001	0.18	0.21
LVOTPG at rest	6.0 (3.0 to 12.3)	6.0 (3.0 to 10.25)	0 (−3.0 to 1.3)	0.023*	6.0 (4.0 to 10.0)	6.0 (4.0 to 11.0)	0.5 (−3.0 to 2.0)	0.025*	0.40*	0.20**
HR, bpm	74.9 ± 15.3	65.8 ± 4.3	−9.1 ± 14.2	<0.001	76.6 ± 16.4	66.6 ± 6.3	−10.0 ± 14.7	<0.001	0.68	0.062
FPG, mmol/L	6.7 ± 2.6	6.1 ± 1.6	−0.58 ± 2.1	0.008	6.2 ± 2.1	6.1 ± 1.4	0.15 ± 1.5	0.33	0.007	0.40
Creatinine, mg/dL	95.1 ± 23.4	97.4 ± 24.3	2.3 ± 15.7	0.16	91.7 ± 35.1	92.1 ± 41.3	0.3 ± 15.4	0.84	0.38	0.13
BNP, pg/mL	424.7 (205.0 to 919.7)	270.5 (125.8 to 419.4)	0 (−607.5 to 0)	<0.001*	191.2 (46.1 to 512.0)	126.0 (35.6 to 334.2)	0 (−105.7 to 0)	<0.001*	0.023*	0.25**
NYHA class	3 (2 to 3)	2 (2 to 2)	−1 (−1 to −0.25)	<0.001*	2 (2 to 3)	2 (1 to 2)	−1 (−1 to 0)	<0.001*	0.031*	0.22**
HF readmission	6 (6.4%)				4 (4.3%)				0.71	0.096

GLT-2i, sodium glucose cotransporter-2 inhibition; HR, heart rate; FPG, fasting plasma glucose; BNP, B-type natriuretic peptide; IVST, Interventricular septal thickness; LAD, left atrial diameter; LVEDD, left ventricular end diastolic diameter; LVEF, left ventricular ejection fraction; E, early diastolic mitral inflow velocity; *e*′, mitral annular tissue velocity; LVOTPG, left ventricular outflow tract pressure gradient; HF, heart failure.

* *Z* value by Mann–Whitney *U* test.

** δ value by Cliff's Delta.

**Figure 3:**
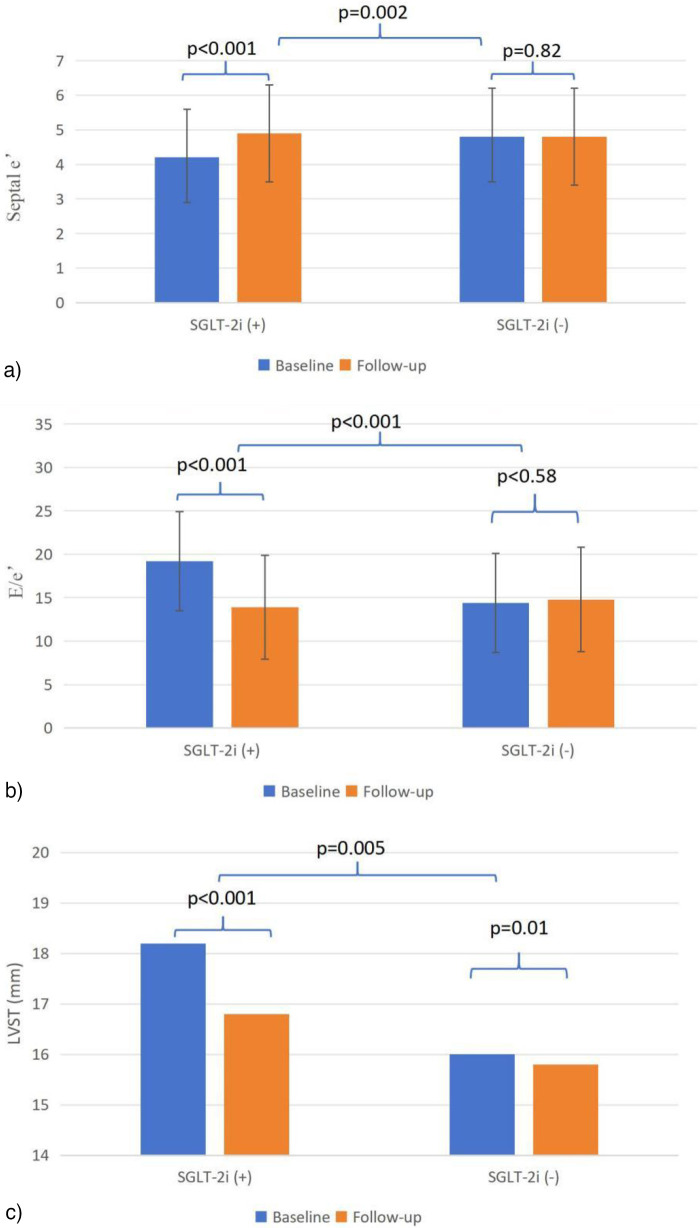
**(a–c)** Echocardiographic parameters from baseline to follow-up. *e*′, mitral annular tissue velocity; *E*, early diastolic mitral inflow velocity; IVST, Interventricular septal thickness.

When compared to the control group, the SGLT-2i group exhibited more pronounced improvements in septal *e*′ (Δ0.7 ± 1.3 vs. Δ0.04 ± 1.6, *p* = 0.002) and *E*/*e*′ (Δ–5.1 ± 8.7 vs. Δ 0.4 ± 6.4, *p* < 0.001), as illustrated in Figures 3a,b. Similarly, greater reductions were noted in IVST [Δ–1.3 (–3.1 to 0) mm vs. Δ −0.2 (–2.0 to 0.9) mm, *p* = 0.005; Figure 3c], NYHA class [Δ −1(−1 to −0.25) vs. Δ −1 (−1 to 0), *p* = 0.031] and BNP levels [Δ0 (–607.5 to 0) pg/mL vs. Δ 0 (–105.7 to 0) pg/mL, *p* = 0.023].

Multivariate analysis, after adjustment for comorbidity of HF, baseline BNP, LAD, LVEF, septal *e*′, *E*/*e*′, use of MRA and loop diuretic (Table 3), confirmed that the improvements in septal *e*′ (*t* = 2.26, *p* = 0.025), *E*/*e*′ (*t* = −3.75, *p* < 0.001), and NYHA class (*p* = 0.038) remained significantly greater in the SGLT-2i group compared to the control group.

**Table 3:** Multivariable analysis.

Parameter	SGLT-2i(+) (*n* = 94)	SGLT-2i(−) (*n* = 94)	*t*	Adjusted difference between groups (95% CI)	*P* value	*R*^2^ values	DW
Δseptal *e*′	0.7 ± 1.3	0.04 ± 1.6	2.26	0.059 to 0.86	0.025	0.31	1.96
Δ*E*/*e*′	−5.1 ± 8.7	0.4 ± 6.4	−3.75	−4.96 to −1.54	<0.001	0.57	2.27
ΔIVST^a^	−1.3 (−3.1 to 0)	−0.2 (−2.0 to 0.9)	–	−1.66 to 0.57	0.34	0.007	2.17
ΔLAD	−1.1 ± 4.5	0.08 ± 5.5	0.18	−1.37 to 1.63	0.86	0.16	1.92
ΔLVEF	1.3 ± 8.8	2.9 ± 6.7	−1.96	−4.14 to 0.019	0.052	0.33	2.19
ΔLVEDD	−0.3 ± 5.5	0.4 ± 6.5	0.036	−1.88 to 1.95	0.97	0.042	2.20
ΔLVOTPG at rest^a^	0 (−3.0 to 1.3)	0.5 (−3.0 to 2.0)	–	−2.13 to 10.67	0.28	0.026	2.09
ΔBNP^a^	0 (−607.5 to 0)	0 (−105.7 to 0)	–	−167.34 to 16.48	0.13	0.75	2.03
ΔFPG	−0.58 ± 2.1	0.15 ± 1.5	−1.63	−1.08 to 0.10	0.11	−0.009	2.00
ΔNYHA class^a^	−1 (−1 to −0.25)	−1 (−1 to 0)	–	−0.50 to −0.018	0.038	0.008	2.11

HF, heart failure; BNP, B-type natriuretic peptide; MRA, mineralcorticoid recept antagonist; LAD, left atrial diameter; LVEF, left ventricular ejection fraction; E, early diastolic mitral inflow velocity; *e*′, mitral annular tissue velocity; SGLT-2i, Sodium glucose cotransporter-2 inhibition; IVST, Interventricular septal thickness; LVEDD, left ventricular end diastolic diameter; LVOTPG, left ventricular outflow tract pressure gradient; FPG, fasting plasma glucose; DW: Durbin-Watson statistic. #No significant multicollinearity was detected, as all variance inflation factors were <5 (HF comorbidity: 1.69, baseline BNP: 1.55, LAD: 1.46, LVEF: 1.19, septal *e*′: 1.47, *E*/*e*′: 1.56, MRA use: 2.45, loop diuretic use: 2.40). (Adjusted for comorbidity of HF, baseline BNP, LAD, LVEF, septal *e*′, *E*/*e*′, use of MRA and loop diuretic#).

^a^ Bootstrap multiple linear regression model.

#### Secondary endpoint

**Figure 4:**
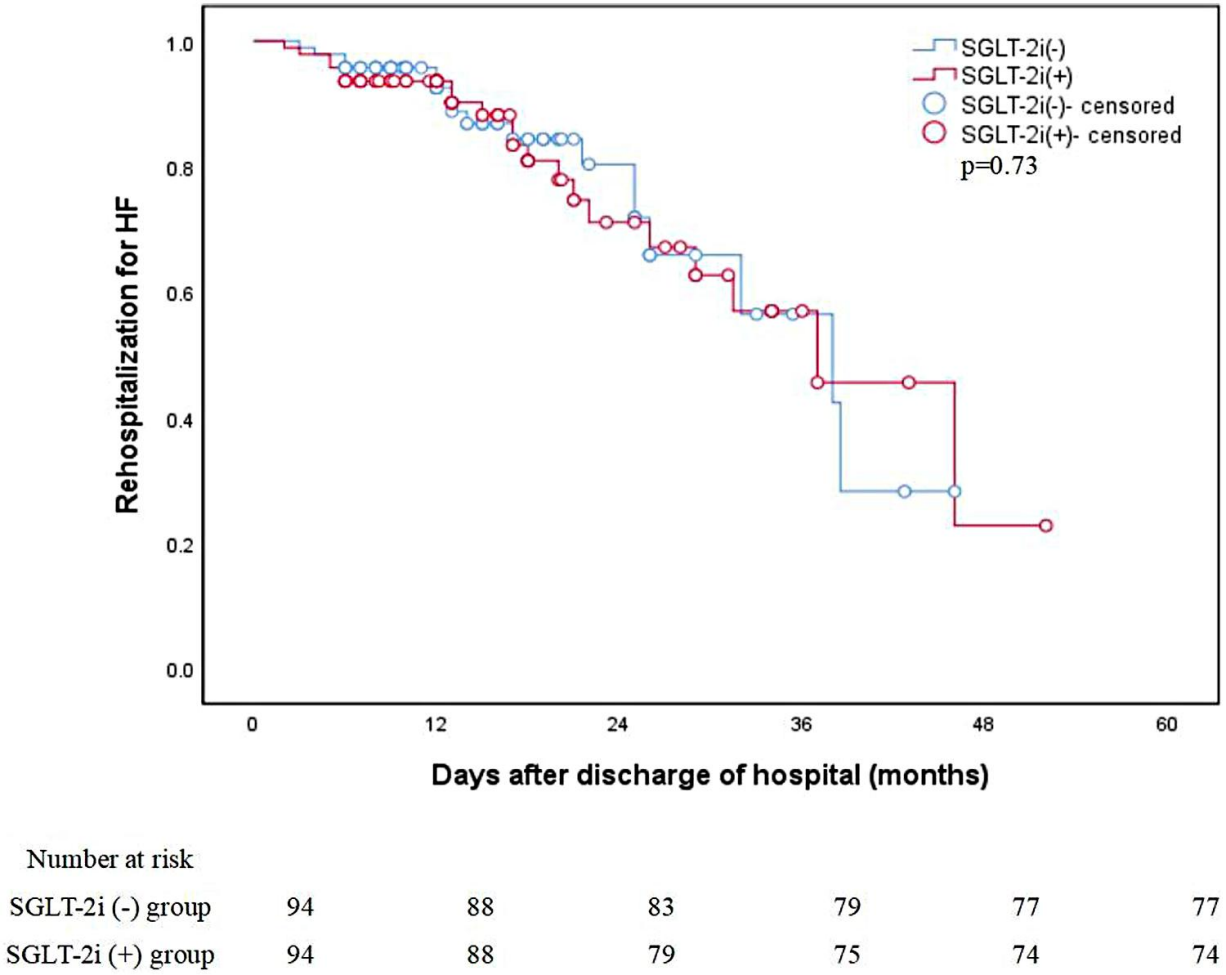
Unadjusted Kaplan–Meier curves: Rehospitalization for HF. HF, heart failure; SGLT-2i, sodium-glucose cotransporter-2 inhibitors.

At the 6-month follow-up, HF readmission occurred in 6 patients in the SGLT-2i group and 4 in the control group (*p* = 0.71) (Table 2). Over a median follow-up of 16.3 months, the Kaplan–Meier analysis demonstrated no significant difference in HF readmission between the two groups (20 events in the SGLT-2i group vs. 17 in the control group; log-rank *p* = 0.73), as illustrated in Figure 4.

#### Safety endpoints

No hypoglycemia and urinary tract infection events were recorded in the patients' outpatient visits and telephone follow-up. And no significant difference in the changes of creatinine (2.3 ± 15.7 vs. 0.3 ± 15.4 mg/dL, inter-group *p* = 0.38) was observed during follow-up between groups (Table 2).

#### Subgroup analysis

A subgroup of 24 oHCM patients with resting LVOTO (13 receiving SGLT-2i and 11 controls) was analyzed. As shown in Table 4, SGLT-2i treatment was associated with a significant improvement in septal *e*′ compared with the control group [1.2 (–0.2 to 1.6) vs. −0.2 (–1.0 to 0.7), *p* = 0.018]. However, no significant between-group difference was observed in the change of resting LVOTPG [–21.0 (–35.5 to −8.0) vs. −28.0 (–61.0 to −13.0), *p* = 0.19].

**Table 4:** Subgroup analysis of oHCM and noHCM at Low-risk.

Parameter	oHCM subgroup				noHCM subgroup without DM or HF			
	SGLT-2i (+) (*n* = 13)	SGLT-2i (−) (*n* = 11)	*Z* value	*p*	SGLT-2i (+) (*n* = 4)	SGLT-2i (−) (*n* = 25)	*Z* value	*p*
Δseptal *e*′	1.2 (−0.2 to 1.6)	−0.2 (−1.0 to 0.7)	2.35*	0.02	1.9 (1.7 to 3.0)	0.3 (−0.8 to 0.9)	2.66*	0.005
Δ*E*/*e*′	−3.4 (−22.0 to 0.02))	−0.5 (−5.8 to 2.7)	−1.59*	0.12	−7.3 (−13.5 to −0.5)	1.7 (−0.2 to 3.8)	−2.77*	0.003
ΔIVST	−2.2 (−4.0 to −0.4)	−2.0 (−3.5 to 1.3)	−0.75*	0.46	−1.7 (−4.4 to 2.0)	0 (−1.8 to 1.1)	−0.64*	0.56
ΔLAD	−1.2 (−2.6 to 2.1)	−0.5 (−4.2 to 3.0)	−0.12*	0.91	−2.4 (−4.6 to −1.4)	0.9 (−2.6 to 3.0)	−1.65*	0.10
ΔLVOTPG at rest	−21.0 (−35.5 to −8.0)	−28.0 (−61.0 to −13.0)	1.36*	0.19	1.0 (−1.0 to 6.0)	1.0 (−2.0 to 2.0)	0.99*	0.35

oHCM, obstructive hypertrophic cardiomyopathy; noHCM, non-obstructive hypertrophic cardiomyopathy; DM, diabetes mellitus; HF, heart failure; E, early diastolic mitral inflow velocity; *e*′, mitral annular tissue velocity; IVST, interventricular septal thickness; LAD, left atrial diameter; LVOTPG, left ventricular outflow tract pressure gradient.

* *Z* value by Mann–Whitney *U* test.

Among the 188 included HCM patients, only 29 did not have comorbid DM or HF, and among these, only 4 were treated with SGLT-2i. In this small subgroup, patients receiving SGLT-2i showed significantly greater improvement in septal *e*′ [1.9 (1.7 to 3.0) vs. 0.3 (–0.8 to 0.9), *p* = 0.005] and *E*/*e*′ [–7.3 (–13.5 to −0.5) vs. 1.7 (–0.2 to 3.8), *p* = 0.003] compared with controls (Table 4).

## Discussion

To our knowledge, this represents one of the first PSM studies to systematically evaluate SGLT-2i specifically in a dedicated cohort of patients with HCM, with particular emphasis on clinically relevant subgroups, including those with LVOTO. Our findings extend the potential therapeutic utility of this drug class from the well-established HF populations to the distinct context of HCM. Notably, we demonstrate that SGLT-2i treatment is associated with a concordant improvement across a triad of disease-specific parameters: a trend towards reduction in IVST, enhancement of early diastolic relaxation (septal *e*′), and a significant decrease in left ventricular filling pressure (*E*/*e*′). This pattern of “structure-function-hemodynamics” benefit was observed in the overall population as well as in key subgroups, without compromising renal function or increasing hypoglycemic risk, and suggests a targeted impact on the core pathophysiology of HCM.

Given that current standard therapies for HCM often leave many patients with persistent symptoms (2), SGLT-2i present a novel therapeutic opportunity. Their pleiotropic effects—including benefits on myocardial energetics, calcium handling, and reverse remodeling (10, 13, 14)—directly target the pathological hallmarks of HCM (1, 2), such as myocardial fibrosis (15) and diastolic dysfunction (16), providing a mechanistic rationale for the improvements observed in our study.

### LV hypertrophy and diastolic function

HCM symptoms primarily stem from LV diastolic dysfunction due to hypertrophy and fibrosis, a pathology shared with HFpEF (3). Initial clinical evidence from Subramanian et al. (17) in non-obstructive HCM patients and supporting preclinical data (18) indicate that SGLT-2i can improve diastolic function, aligning with our findings. Furthermore, SGLT-2i may mitigate key pathological processes like myocardial fibrosis and promote reverse remodeling (10, 13–16). The DAPA-LVH trial (15) demonstrated that dapagliflozin significantly reduced left ventricular mass in patients with diabetes and LV hypertrophy, confirming its capacity to reverse this key structural abnormality.

This study provides evidence of a multi-dimensional and physiologically coherent benefit associated with SGLT-2i therapy in HCM (17). Although the absolute changes in some echocardiographic parameters were modest, their clinical relevance must be interpreted within the context of a chronic, slowly progressive disease where reversal of cardiac abnormalities is inherently limited (1, 3). The minimal change observed in the control group aligns with this expected natural history (1). In contrast, the SGLT-2i group demonstrated a concordant pattern of improvement: a trend toward structural change with reduced IVST (ΔIVST: −1.3 vs. −0.2 mm), a significant enhancement in early diastolic relaxation velocity (Δ septal *e*′: 0.7 vs. 0.04 cm/s), and a marked reduction in left ventricular filling pressure (Δ*E*/*e*′: −5.1 vs. 0.4). These parameters are physiologically interlinked, suggesting that attenuation of hypertrophy (15) may facilitate improved relaxation, which in turn lowers filling pressures (16, 17). Notably, the improvement in *E*/*e*′ was substantial (Cohen's *d* = 0.72), a parameter with direct clinical relevance to symptoms and prognosis in HCM (3). The coherent “structure-function-hemodynamics” improvement observed in this study suggests that SGLT-2i may modulate the pathological milieu in HCM through pathways such as improved myocardial energetics and attenuated fibrosis. This pharmacological strategy intriguingly resonates with fundamental discoveries in cardiovascular remodeling—for instance, recent work highlighting the pivotal role of signaling axes like MrgD/PIM1 in regulating pathological hypertrophy and fibrosis (19). While the specific molecular targets of SGLT-2i differ, they share the core biological principle of modulating the adverse cellular and extracellular matrix environment to facilitate functional recovery (19, 20).

A nuanced interpretation of the structural data remains warranted, however. Specifically, the between-group difference in IVST reduction did not reach statistical significance after multivariable adjustment. This likely reflects the biological reality that structural reversal lags behind functional improvement (16), especially in HCM where hypertrophy is a fundamental, genetically driven pathology and a particularly challenging therapeutic target (2, 3). Therefore, the consistent trend observed here should be regarded as an important preliminary signal, providing a rationale for investigating whether longer-term SGLT-2i treatment can translate these early functional benefits into clinically meaningful structural remodeling, thereby potentially interrupting the cascade from hypertrophy to overt HF.

Thus, our findings not only support the potential disease-modifying value of SGLT-2i in HCM but also situate it within the broader therapeutic endeavor to reverse maladaptive cardiac remodeling. This holistic evidence base strengthens the therapeutic rationale for SGLT-2i in HCM beyond isolated parameter changes.

### Cardiovascular events

Large real-world studies support SGLT-2i's benefits in HCM. A TriNetX analysis (21) of 872 matched patients showed a 76% lower all-cause mortality and 37% fewer cardiovascular events with SGLT-2i after two years. Similarly, a Korean cohort study (22) of 4,126 HCM patients with diabetes associated SGLT-2i use with significantly reduced risks of heart failure hospitalization (18%), all-cause mortality (45%), and sudden cardiac death (50%). These cohorts had high comorbidity rates, aligning with established high-risk profiles in HCM (23).

In our study, most patients initiated SGLT-2i due to comorbid DM or HF, which align with its approved clinical indications (8). It is noteworthy that the observed significant improvements in LV diastolic function and filling pressure did not translate into a significant reduction in HF hospitalization over a median follow-up of approximately 16.3 months in this study. This finding warrants careful interpretation within the context of the study design and disease pathophysiology. First, the follow-up duration may have been insufficient. In large randomized trials (11, 24) of SGLT-2i in HFpEF (a phenotype akin to HCM-related HF), the benefit on HF hospitalization typically requires more than 2 years to fully emerge. The early functional and structural improvements captured in our study may thus represent a potential harbinger of longer-term clinical benefit. Second, HF hospitalization is a multifactorial composite endpoint, particularly influenced in HCM by complex mechanisms such as dynamic outflow tract obstruction and arrhythmias (1, 2). The statistical power of our study, given its sample size and follow-up period, was limited for detecting potentially modest differences in this endpoint. Finally, this aligns with the recognized mode of action of SGLT-2i—the biological benefits mediated through improved myocardial energetics, reduced fibrosis, etc., are progressive and cumulative (10, 13, 14). Consequently, a temporal dissociation between short-term functional improvement (a “disease-modifying” signal) and a future reduction in hard endpoints is plausible. Future studies with longer follow-up, larger sample sizes, and possibly incorporating intermediate clinical endpoints such as exercise capacity, are needed to fully elucidate the long-term clinical value of SGLT-2i in HCM.

### LVOTO and therapeutic implications

LVOTO is a key determinant of symptoms in oHCM (25). While first-line therapies like beta-blockers or myosin inhibitors (2, 5, 6, 26) primarily aim to reduce the gradient, our study found that SGLT-2i did not confer additional reduction in LVOT gradient compared to controls. This important finding helps define the therapeutic niche of SGLT-2i: its benefit in HCM appears largely independent of mechanical relief of obstruction.

Consequently, in oHCM, SGLT-2i should be viewed not as a treatment for obstruction, but as a potential adjunctive therapy targeting the underlying myocardial pathophysiology (e.g., diastolic dysfunction, fibrosis) (10, 13, 14), particularly in patients with concomitant HF or DM. For the substantial population with noHCM, where specific disease-modifying drugs are scarce, the improvements in diastolic function and structure observed in our study suggest SGLT-2i may hold particular promise, warranting dedicated investigation.

### Arrhythmia

Arrhythmias, particularly AF, are major complications in HCM, driving HF and stroke risk (1, 2). The SHaRe registry (*N* = 4,591) reported high incidence rates of AF (20%) and ventricular arrhythmias (6%) in HCM cohort (27, 28). While guidelines recommend anticoagulation for all HCM patients with AF, SGLT-2i has been associated with a 26% reduction in ischemic stroke (26), though a large meta-analysis found it did not significantly lower AF incidence (29).

Ventricular arrhythmias, a leading cause of sudden cardiac death (SCD), stem from structural and electrical remodeling in HCM (1). SGLT-2i may mitigate this risk by attenuating fibrosis, reversing adverse remodeling, and stabilizing electrical activity through mechanisms such as improved myocardial energetics and calcium handling (10, 13–15, 30). Clinical trials in HF populations have demonstrated that SGLT-2i significantly reduces ventricular arrhythmias (31, 32), including sustained ventricular tachycardia/ventricular fibrillation recorded in patients with ICDs (32).

Critically, a recent meta-analysis (33), extended this benefit to SCD prevention. This meta-analysis by Matteucci et al., encompassing 58,569 patients across 8 RCTs, demonstrated that SGLT-2i significantly reduced the risk of SCD (OR: 0.82; 95% CI: 0.72–0.94; *P* = 0.0104) in broad populations with DM, HF, or CKD, thereby expanding the spectrum of their cardiovascular benefits. The prevention of SCD remains a major unmet clinical challenge, especially in individuals without high-risk features amenable to device therapy, as pharmacological strategies specifically approved for SCD primary prevention are currently lacking. In this context, the emerging evidence for SGLT-2i (33), alongside their proposed mechanisms of attenuating fibrosis, reversing adverse remodeling, and potentially stabilizing cardiomyocyte electrophysiology (10, 14), represents a paradigm-shifting advance in arrhythmic risk management. Although not yet studied specifically in HCM, SGLT-2i represents a particularly promising therapeutic option to address the residual risk of SCD in this population.

### Adverse events

SGLT-2i demonstrate a favorable safety profile in heart failure and diabetes populations. While known class effects include urinary tract infections and diabetic ketoacidosis, a large meta-analysis (34) confirmed no significant increase in hypoglycemia or fracture risk. Real-world data from the TriNetX study (19) and our findings support its overall safety and good tolerability in HCM patients with clinical indications. Expert consensus (35) emphasizes patient evaluation and education prior to initiation.

### Future perspectives

This study provides real-world evidence for the benefit of SGLT-2i in HCM using conventional clinical imaging and functional biomarkers. Looking forward, advancing this field will require a deeper mechanistic understanding and dynamic monitoring of the myocardial microenvironment. Emerging biosensing technologies—such as electrochemical methods for the real-time detection of mitochondrial oxidative stress markers (36)—exemplify the next generation of tools for precise risk stratification and therapy monitoring in cardiomyopathy. Integrating such precision biomarkers with traditional endpoints in future research is a crucial step toward personalized and dynamic management of HCM.

### Limitations

Despite the findings presented, this study has several limitations that should be acknowledged: (1) Selection bias: As a single-center retrospective analysis, the study included a high proportion of patients with multiple comorbidities, many of whom were already on guideline-directed therapies for HF or diabetes. This may limit the generalizability of the results, particularly to low-risk HCM populations, which were underrepresented in our cohort. (2) Short follow-up duration: The relatively short follow-up period precludes definitive conclusions regarding the long-term efficacy and safety of SGLT-2i in HCM. (3) Unmeasured medication adherence: As a retrospective study, direct monitoring of adherence (e.g., via pill count) was not feasible. Adherence was assessed indirectly using available clinical data, including the frequency of prescription refills in outpatient records and physician documentation of patient-reported medication intake during follow-up visits. (4) Residual confounding: Despite statistical adjustments, unmeasured or unknown confounders inherent to observational designs may affect the results. (5) Data completeness: Retrospective data collection is susceptible to missing or inconsistently recorded information, potentially introducing bias. (6) Limited power for subgroup analyses: The small sample size in certain subgroups (e.g., low-risk patients) reduces the reliability of subgroup-specific conclusions.

## Conclusion

In this real-world, PSM study, initiation of SGLT-2i therapy was associated with significant improvements in LV diastolic function and NYHA functional class in patients with HCM, without increasing renal or hypoglycemic risk. These findings support the potential therapeutic value of SGLT-2i, particularly in HCM patients with comorbidities like HF or diabetes where these agents are already indicated. However, given the observational design and single-center nature of our study, large-scale, prospective randomized trials are warranted to confirm efficacy, identify optimal patient subgroups, and elucidate underlying mechanisms before broader clinical recommendations can be made.

## Data Availability

The raw data supporting the conclusions of this article will be made available by the authors, without undue reservation.
